# Integrated structural biology of the native malarial translation machinery and its inhibition by an antimalarial drug

**DOI:** 10.1038/s41594-025-01632-3

**Published:** 2025-08-18

**Authors:** Leonie Anton, Wenjing Cheng, Meseret T. Haile, Jerzy M. Dziekan, David W. Cobb, Xiyan Zhu, Leyan Han, Emerson Li, Anjali Nair, Carolyn L. Lee, Hanyu Wang, Hangjun Ke, Guoan Zhang, Emma H. Doud, Alan F. Cowman, Chi-Min Ho

**Affiliations:** 1 Department of Microbiology and Immunology, Columbia University Irving Medical Center, New York, NY USA; 2 Department of Physiology and Cellular Biophysics, Columbia University Irving Medical Center, New York, NY USA; 3 The Walter and Eliza Hall Institute of Medical Research, Parkville, Victoria Australia; 4 Department of Microbiology and Immunology, Drexel University College of Medicine, Philadelphia, PA USA; 5 Proteomics and Metabolomics Core Facility, Weill Cornell Medicine, New York, NY USA; 6 Center for Proteome Analysis, Indiana University School of Medicine, Indianapolis, IN USA; 7 University of Melbourne, Melbourne, Victoria Australia; 8 Present Address: Institute of Biochemistry and Molecular Medicine, University of Bern, Bern, Switzerland; 9 Present Address: Newborn Screening and Molecular Biology Branch, Centers for Disease Control, Atlanta, GA USA

**Keywords:** Cryoelectron tomography, Cryoelectron microscopy, Parasitology, Drug discovery

## Abstract

Our understanding of cellular events is hampered by the gap between the resolution at which we can observe events inside cells and our ability to replicate physiological conditions in test tubes. Here, we show in *Plasmodium falciparum*, a non-model organism of high medical importance, that this gap can be bridged by using an integrated structural biology approach to visualize events inside the cell at molecular resolution. We determined eight high-resolution structures of the native malarial ribosome in actively translating states inside *P.* *falciparum*-infected human erythrocytes using in situ cryo-electron tomography. Following perturbation with a *Plasmodium*-specific translation inhibitor, we then observed a decrease in elongation factor-bound ribosomal states and an apparent upregulation of ribosome biogenesis in inhibitor-treated parasites. Our work elucidates new molecular details of the malarial translation elongation cycle and demonstrates direct multiscale visualization of drug-induced phenotypic changes in the structure and localization of individual molecules within the native cellular context.

## Abstract (web summary)

Integrated structural biology approach leveraging in situ cryo-electron tomography reveals molecular details of the native malarial translation elongation cycle and its perturbation by cabamiquine, a potent antimalarial drug candidate.

## Main

Understanding the molecular consequences of perturbations from compounds, mutations or stress on cells is a fundamental aspect of basic cell biology research and an essential component of drug discovery^1,2^. This is currently approximated using in vivo cell-based assays and, if the molecular target is known, in vitro biochemical and biophysical assays^3^. The information we glean from the former, while physiological, is coarse-grained, comprising primarily gross morphological changes on the scale of cells or even tissues. Conversely, the latter provides insight into molecular mechanisms but is devoid of physiological context, often failing to reflect what happens inside the cell. This major gap hinders our ability to fully understand the molecular basis of cellular responses to perturbations and contributes to high attrition and low success rates in the development of new therapeutics^1,2,4,5^.

Recent advances in cellular cryo-electron tomography (in situ cryo-ET) have the potential to bridge this gap by enabling multiscale visualization of the cell, providing atomic-resolution details of molecular structures under physiological conditions, as well as their organization within supramolecular assemblies and cell-wide ultrastructures in cryopreserved cells^6–10^. This technique has been used to visualize native 80S ribosomes in various steps of translation elongation^11,12^, in one case bound to a well-characterized ribosome inhibitor with an established binding site and mode of action^13^. However, because of the challenging nature of this nascent technique, these important proof-of-principle studies have thus far been limited to well-studied model organisms.

Building upon these important proof-of-principle studies in ideal systems, we use in situ cryo-ET to reveal the native translation elongation cycle and its perturbation by a species-specific translation inhibitor in a non-model organism of high medical importance: the malaria-causing parasite *Plasmodium falciparum*. Malaria exacts a devastating economic and public health burden that is exacerbated by the persistent rise of resistance to frontline therapeutics, posing an urgent need for new antimalarials^14^. Consequently, where previous studies have demonstrated in situ visualization of ribosomes with established broad-spectrum ribosomal inhibitors, we aimed to shed light on the molecular effects of a malaria-specific translation inhibitor currently in phase 2 clinical trials with an unknown mode of action^15^. *P.* *falciparum* is a complex non-model organism with unique biology, much of which remains poorly understood because of the exceptional challenges it presents to genetic manipulation, recapitulation for study in recombinant systems and structural studies. As such, unlike the model organisms characterized in previous in situ cryo-ET studies, purified *Pf*80S ribosomes have not been extensively studied in vitro or in vivo and the molecular details of malarial translation remain largely unknown.

Here, we present nine new native structures of the *Pf*80S ribosome, including eight active translation intermediate states, determined using in situ cryo-ET of *P.* *falciparum-*infected human erythrocytes, which elucidate the molecular details of the malarial translation elongation cycle. We then visualize how the addition of a *Plasmodium*-specific translation inhibitor perturbs this cycle at molecular resolution in parasite-infected human erythrocytes, demonstrating phenotypic characterization of an antimalarial compound within the native physiological context at unprecedented resolutions using in situ cryo-ET, intact cell proteome integral solubility assays (PISAs) and proteomics. Taken together, our data represent a major advance in resolution of observable phenotypic changes in the cell in response to perturbation in the native cellular context.

## Results

### In vitro structures (2.4–2.8 Å) of the *Pf*80S ribosome

**Fig. 1:**
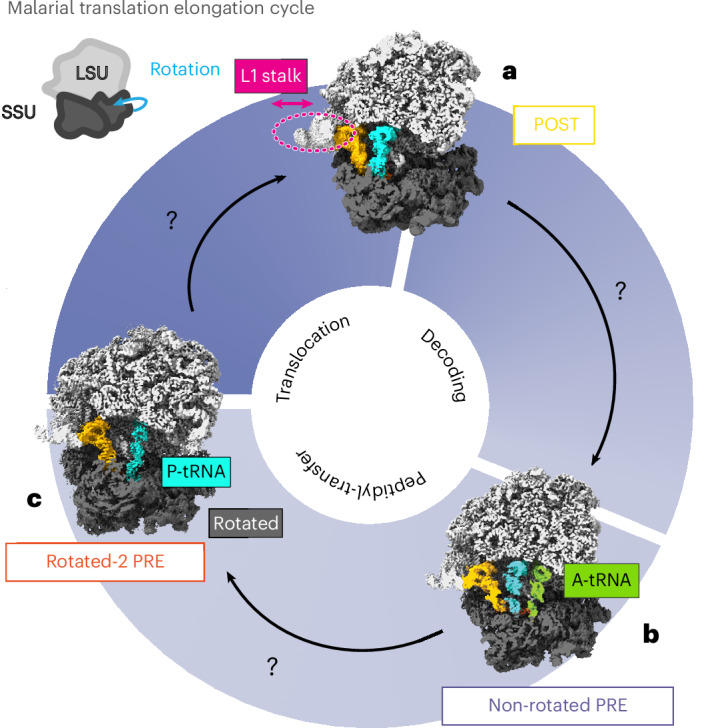
Single-particle *Pf*80S ribosomes. **a**–**c**, Single-particle cryo-EM reconstructions, at 2.4–2.8-Å resolution, of the *Pf*80S ribosome in the POST state with P-site and E-site occupied, with the L1 stalk indicated (**a**), non-rotated PRE state with P-site, E-site and A-site occupied (**b**) and rotated PRE state with P-site and E-site occupied (**c**). The map is colored as follows: 60S large subunit, light gray; 40S small subunit, dark gray; A-site, P-site and E-site tRNAs, green, cyan and yellow, respectively. LSU, large subunit; SSU, small subunit.

**Extended Data Fig. 1:**
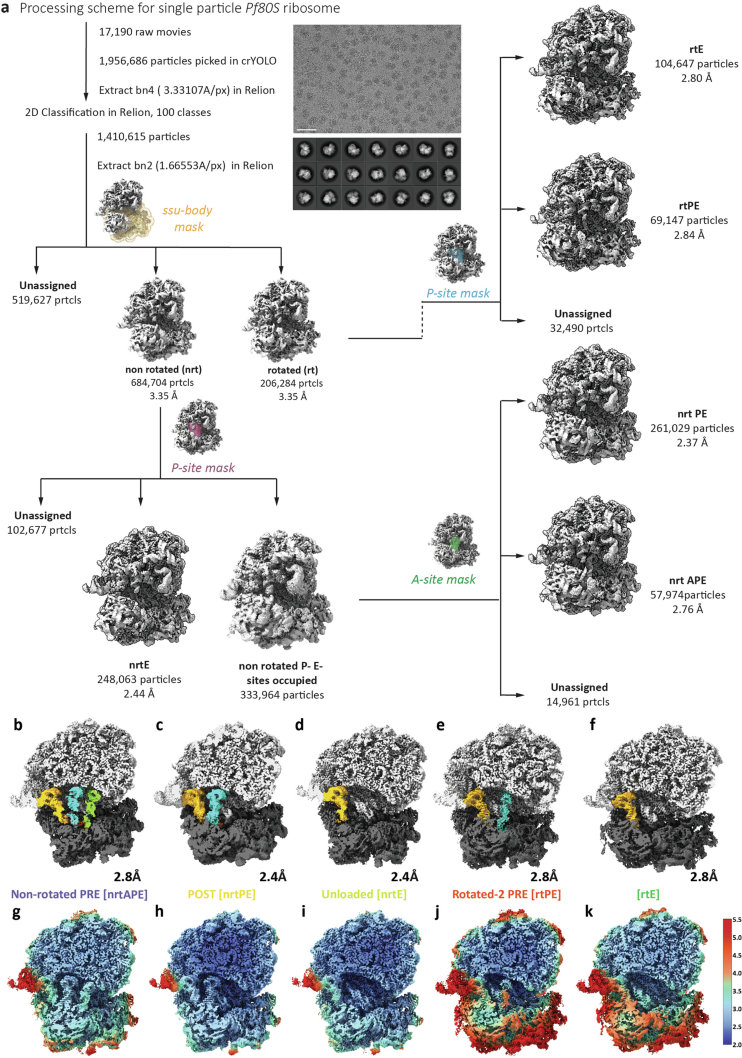
Single particle data processing scheme and refined structures of the *Pf*80S ribosome. **a**, Five states were separated as displayed in the pictured classification scheme, using the following masks: small-subunit (yellow) for separating rotated from non-rotated states, P-site (pink) for non-rotated particles, P-site (blue) for rotated particles and A-site (green) for non-rotated particles. **b-f**, Final post-processed maps of the five *Pf*80S ribosome states, shown in cross section. Maps are colored as follows: 60S large subunit in light grey, 40S small subunit in dark grey, mRNA in dark orange, and the A-, P-, and E-site tRNAs in green, cyan, and yellow, respectively. **g-k**, Corresponding cross-sectioned view of each *Pf*80S map is shown colored according to local resolution, as calculated in RELION.

Despite the size and abundance of ribosomes, achieving sufficient resolution in subtomogram-averaged (STA) in situ cryo-ET reconstructions to unambiguously distinguish different translation intermediates is extremely challenging. To date, there are only five unique published STA in situ cryo-ET maps of eukaryotic 80S ribosomes below 5 Å (three consensus maps and only two active translation intermediates), seven at 4–5-Å resolution and seven at 5–8-Å resolution, all from model organisms^11–13,16^. As there are only two published cryo-electron microscopy (cryo-EM) structures of the *Pf*80S in active translation intermediate states^17^, at moderate resolutions of 6.7 and 7.0 Å, we used single-particle cryo-EM to obtain in vitro structures from purified *Pf*80S ribosomes, yielding five distinct states at substantially higher overall resolutions of 2.4–2.8 Å (Fig. 1 and Extended Data Fig. 1). Our structures include three active translation intermediate structures, non-rotated *Pf*80S with P-site and E-site transfer RNAs (tRNAs), non-rotated *Pf*80S with A-site, P-site and E-site tRNAs, and rotated *Pf*80S with P-site^16^ and E-site tRNAs (Fig. 1), as well as two off-pathway structures, non-rotated *Pf*80S with an E-site tRNA and rotated *Pf*80S with an E-site tRNA (Extended Data Fig. 1d,f).

### Elucidating the native malarial translation elongation cycle

**Fig. 2:**
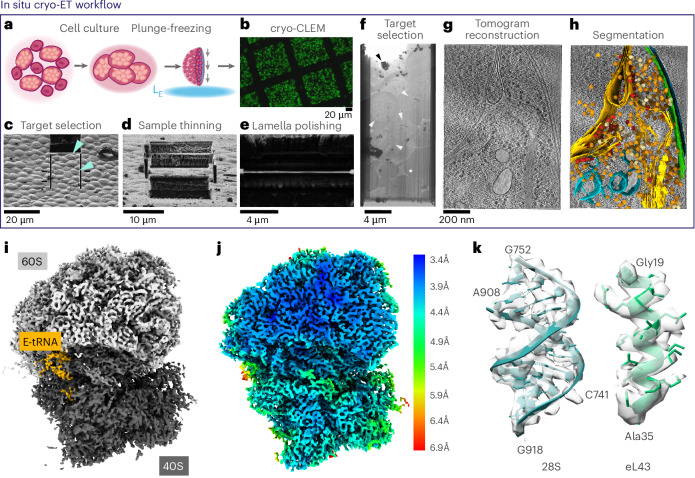
Visualizing molecular details of *P.* *falciparum* asexual intraerythrocytic life-cycle stages using in situ cryo-ET. **a**,**b**, In situ cryo-ET workflow from sample preparation to data analysis. Parasite-infected red blood cells (iRBCs) were applied to a grid support and plunge-frozen in liquid ethane (L_E_) (**a**), followed by cryo-correlative light and EM (CLEM) imaging showing distribution of fluorescently labeled parasites (**b**). **c**, Top-down view of a frozen grid in a cryo-FIB-SEM. Blue arrowheads, schizont iRBCs. **d**,**e**, FIB images of the sample thinned to ~3 µm (**d**) and the final thinned cross-section (lamella) at ~150 nm (**e**). **f**, Montaged cryo-TEM image of the entire lamella. White arrowheads, parasites; black arrowheads, contamination; white star, milling artifacts. **g**, Averaged central slice of a tomogram reconstructed from tilt series collected on lamella. **h**, Ultrastructural segmentations and STA *Pf*80S ribosomes mapped back into the tomographic volume and overlaid onto the corresponding central slice from **g**. **i**, STA consensus reconstruction of the *Pf*80S ribosome at an overall resolution of 4.1 Å. **j**, *Pf*80S consensus map, shown in cross-section and colored according to local resolution, as calculated in RELION3.1. **k**, Ribbon models of 28S rRNA and eL43 ribosomal protein segments, shown with corresponding STA density (surface representation).

**Extended Data Fig. 2:**
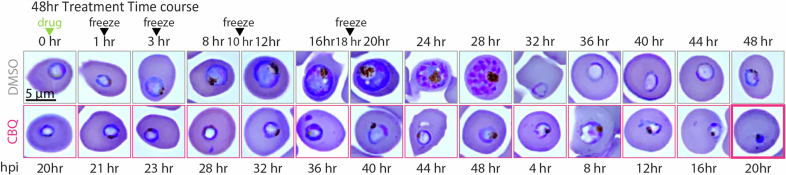
48 h time course following treatment with CBQ at 20hpi. Hema3-stained blood smears of highly synchronized early trophozoite-stage *P. falciparum* parasites, taken at 0, 1, and 3 h after addition of DMSO, CBQ, or CHX at 20hpi, and every 4^th^ hour after, out to 48 h. Three biological replicates were performed, with n = 25-81 for each time point in each replicate. One representative replicate is shown. Time of drug addition at 20hpi and time points corresponding to those used in our *in situ* cryoET studies are indicated with a green and black arrowheads, respectively. Box with a bold pink outline indicates a pyknotic cell.

**Extended Data Fig. 3:**
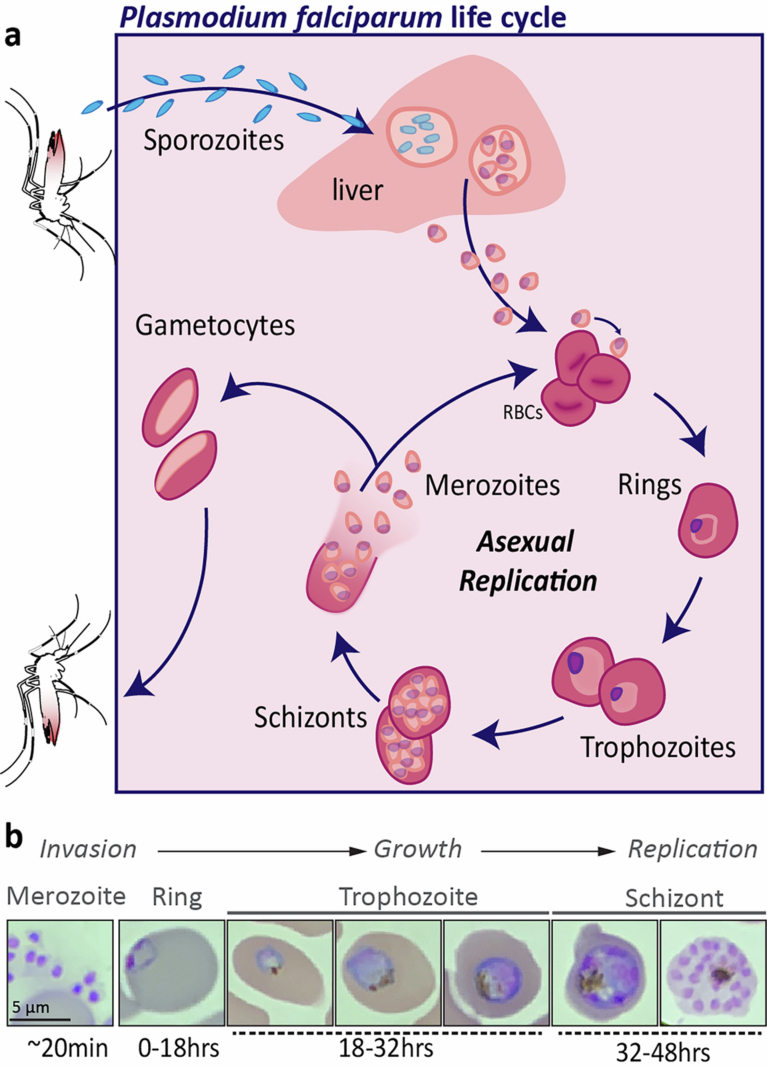
The *P. falciparum* asexual intraerythrocytic life cycle. **a**, Schematic of the *P. falciparum* life cycle, with focus on the intraerythrocytic asexual replication stages in human red blood cells (RBCs). **b**, Representative images, from 12 independent experiments, of Hema3-stained, parasite-infected RBCs (iRBCs) at the different intraerythrocytic stages from invasion to replication drawn from (n = 25-81). Averaged central slice of a tomogram reconstructed from tilt series collected on lamella.

To define the ensemble of compositional and conformational states sampled by malarial ribosomes within the native cellular context and explore how treatment with a *Plasmodium*-specific translation inhibitor affects their distribution among these states, we used cryo-focused ion beam scanning EM (cryo-FIB-SEM) to create 150–200-nm-thin cellular sections (lamellae) of cryo-preserved trophozoite-stage *P.* *falciparum* parasites. The parasites were treated with cabamiquine (CBQ), an established *Plasmodium*-specific translation inhibitor currently in phase 2 clinical trials^18^, or DMSO vehicle at 20 h post invasion (hpi) and isolated 1, 3, 10 or 18 h after addition of the drug (21, 23, 30 or 38 hpi) (Extended Data Fig. 2), as well as untreated parasites at the trophozoite (26–34 hpi), schizont (40–44 hpi) and merozoite stages (Fig. 2a–f and Extended Data Fig. 3). As ring-stage parasites occupy a very small percentage of the total host cell volume, we were unable to obtain sufficient in situ data for downstream analysis from this stage. Instead, we prepared frozen grids containing ribosomes crudely enriched from ring-stage parasites. We collected and analyzed 1,323 in situ tomograms from 117 lamella containing 212 intact cells and 480 tomograms from frozen ex vivo ring-stage parasite lysate (Extended Data Table 2). We then identified and extracted *Pf*80S ribosome particles from all tomograms, yielding 189,474 in situ particles and 28,817 ex vivo particles (a mere 13% of our total dataset).

**Fig. 3:**
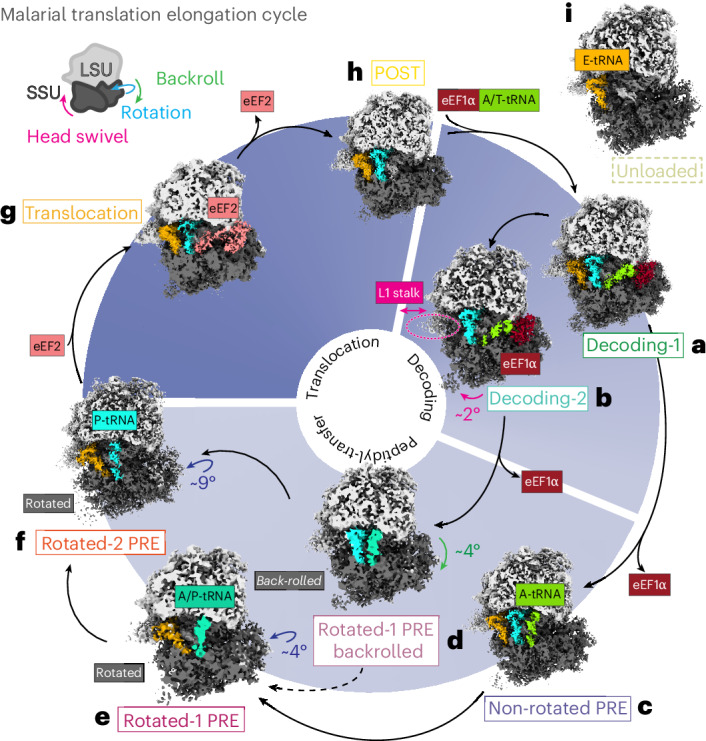
Ensemble of in situ *Pf*80S ribosomes comprising the malarial translation elongation cycle. **a**–**i**, Native structures of the *P.* *falciparum* translational machinery, determined by in situ cryo-ET, elucidate the molecular ensemble of *Pf*80S ribosomes in malaria parasites. Directions of the small subunit rotation, backroll and head swivel are indicated on a cartoon of an 80S ribosome relative to the large subunit. Translation intermediate states (**a**–**h**) and unloaded ribosomal states (**i**) distinguished by position of principal ligands in these high-resolution STA reconstructions, enabling reconstitution of the *P.* *falciparum* translation elongation cycle. The refined map for each state is reconstructed from all particles corresponding to a particular class across datasets. Maps are shown in cross-section for clarity. eEF1α, dark red; A-tRNA, green; L1 stalk movement, pink; A/P-tRNA, teal; P-tRNA, cyan; eEF2, salmon; E-tRNA, orange.

**Extended Data Fig. 4:**
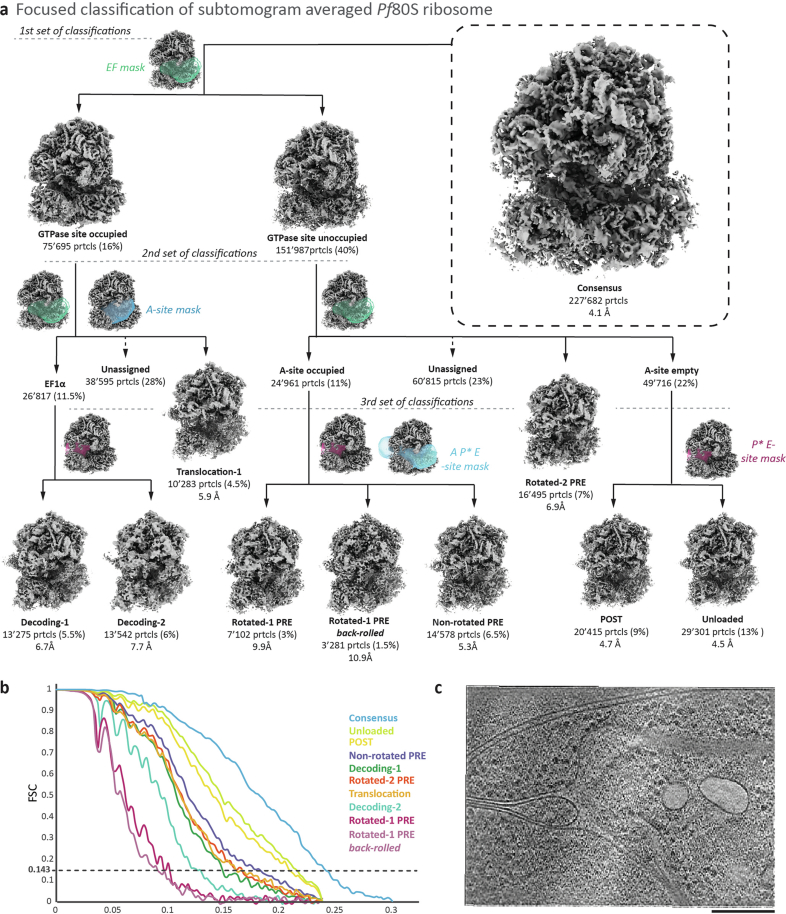
Focused classification of the subtomogram averaged native *Pf*80S from consensus reconstruction. **a**, Translation intermediates were separated following the displayed scheme using the following masks: elongation factor (EF) (green), PE-tRNA (magenta), EF-A-P-PE-E binding sites (cyan), P-tRNA (purple). For all panels the number of particles, percentage of total particles and resolution are indicated below each map. **b**, Fourier shell correlation (FSC) curve for ribosomal states identified through classification to determine global resolution using the ‘Gold-standard’ 0.143 cut off (dotted line). Identified ribosomal states are shown in descending order from highest to lowest resolution in the left side of the graph. **c**, Averaged central slice of a tomogram reconstructed from tilt series collected on lamella.

STA and three-dimensional (3D) refinement on the combined particles (Fig. 2h–k) yielded a consensus structure at an overall resolution of 4.1 Å (Fig. 2j and Extended Data Tables 2 and 4). Resolutions reach the Nyquist limit of 3.4 Å in the core of the large subunit and range from 3.4–4.4 Å throughout most of the map (Fig. 2j). Some lower-resolution (5–10 Å) areas are found at the periphery, particularly in the head region of the 40S small subunit and around the guanosine triphosphatase (GTPase)-binding site, as is expected given that the map consists of an average of the full ensemble of compositional and conformational states sampled by these ribosomes inside the parasite. To separate out all these states, we performed multiple rounds of focused 3D classification on the particles in the consensus structure using masks around the tRNA-binding sites and GTPase-binding sites of the ribosome, yielding a total of nine distinct ribosomal states, comprising ~49% of our dataset (Fig. 3a–i and Extended Data Fig. 4). The remaining 51% of the particles in our datasets could not be readily assigned to any known ribosomal states (Extended Data Fig. 4a). As illustrated in Fig. 3, we assigned eight of our high-resolution states to active translation intermediates comprising the translocation, decoding and peptidyl-transfer phases of elongation (Fig. 3). Notably, five of our eight active translation intermediates have not previously been observed inside eukaryotic cells.

Here, we present the native states observed in situ. Starting with the first step of a cycle of translation elongation, *Pf*eEF1ɑ (elongation factor 1ɑ) delivers a new tRNA into the A-site, bringing the ribosome into the decoding-1 state (Fig. 3a), with all three tRNA sites occupied and *Pf*eEF1ɑ bound in the ribosome GTPase site. Upon proper base pairing, either the E-site tRNA or *Pf*eEF1ɑ dissociates, bringing the ribosome into the decoding-2 state (Fig. 3b) (with tRNAs in the A-site and P-site tRNAs and *Pf*eEF1ɑ still bound) or the non-rotated pre-translocation (non-rotated PRE) state (Fig. 3c) (with tRNAs in the A-site, P-site and E-site and an unoccupied GTPase site), respectively. The A-site and P-site tRNAs then shift as the 40S small subunit either rolls backward or rotates and the nascent peptide is transferred from the P-site tRNA to the A-site tRNA, thereby transitioning the ribosome through the rotated-1 PRE backrolled state (Fig. 3d) (tRNAs in hybrid A/P and P/E positions) or the rotated-1 PRE state (Fig. 3e) (tRNAs in the E-site and a hybrid A/P position), respectively, before entering the rotated-2 PRE (Fig. 3f) with tRNAs in the P-site and E-site. *Pf*eEF2 then enters the GTPase site, rotating the 40S small subunit back to a non-rotated position and bringing the ribosome into the translocation state (Fig. 3g). Translocation then proceeds, shifting the two tRNAs fully into the E-site and P-site and a new codon into the A-site, after which GTP hydrolysis occurs and *Pf*eEF2 dissociates, leaving the ribosome in the post-translocation (POST) state (Fig. 3h), ready for the next cycle of elongation to proceed.

The presence of the decoding-2, rotated-1 PRE backrolled and non-rotated PRE states together in our parasites suggests that dissociation of the E-site tRNA may occur independently of A-site occupancy, explaining why the E-site is only partially occupied both during and after decoding. Furthermore, the dissociation of the E-site tRNA from the non-rotated PRE state alters the energy landscape of the ribosome such that rotation and possibly back-rolling of the small subunit are favored.

**Extended Data Fig. 5:**
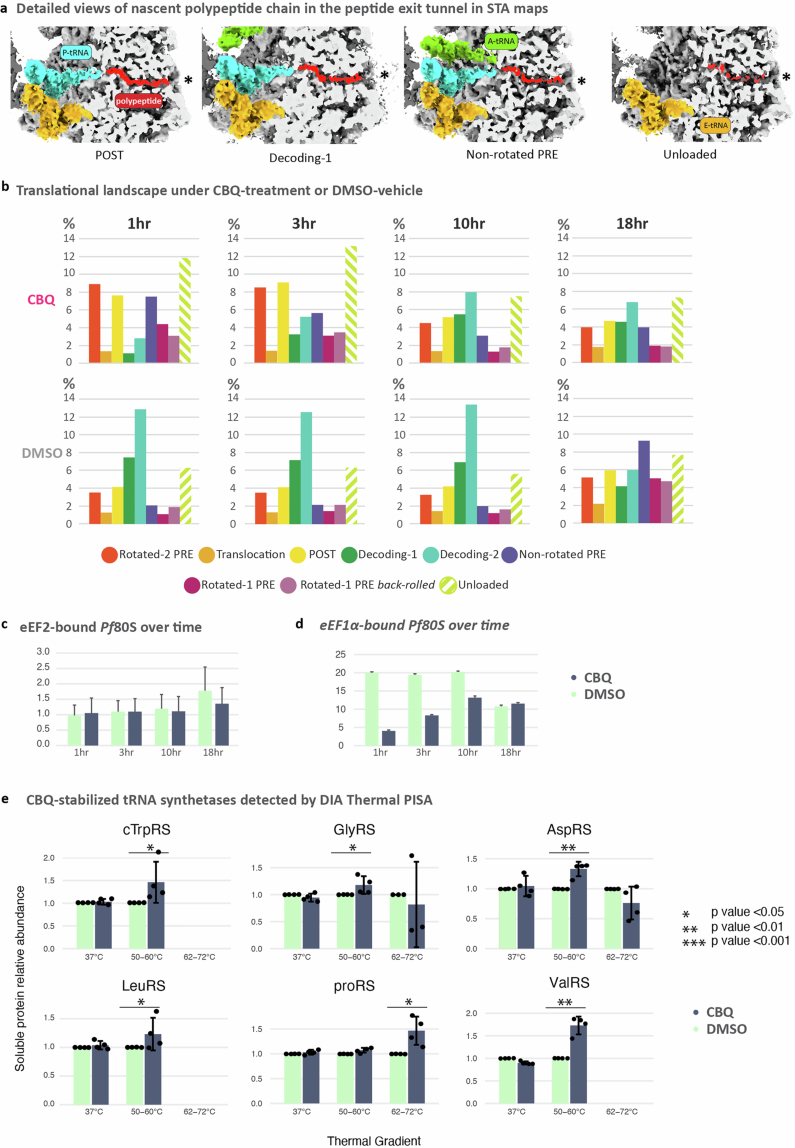
Details of subtomogram averaged maps and perturbations of the translational landscape under CBQ-treatment. **a**, Examples of nascent chain density visible in the exit tunnel of POST, decoding-1 and decoding-2 and unloaded states. **b**, Bar graphs showing the translational landscape comprised of relative distribution of identified translation intermediates and ribosomal states in CBQ-treated and DMSO-control parasites. Percentages are based on total amount of identified ribosomes after particle curation. **c-d**, Bar graph showing the percentage of elongation factor-bound states across 4 time points in CBQ-treated and DMSO- control parasites, across four technical replicates. Percentages are based on total amount of identified ribosomes after particle curation (n = 230,471). Error bars represent SD from the mean. **e**, Relative soluble protein abundance of four CBQ-stabilized tRNA synthetases identified in the DIA Thermal PISA experiment: Arginine tRNA Ligase (ArgRS), Alanine tRNA Ligase (AlaRS), Leucine tRNA Ligase (LeuRS), Tryptophan tRNA Ligase (TrpRS). Protein abundance levels under CBQ treatment are plotted relative to the DMSO vehicle in each respective thermal gradient (n = 4 biological replicates). Significance changes (moderated t-test) are represented by asterisks (*p value* < 0.01 ‘**’; < 0.001 ‘***’). Error bars represent ± SD.

The ninth state, which we term the unloaded state, contains nothing but an E-site tRNA and does not fit into the classical translational cycle (Fig. 3i). We see strong nascent peptide chain density in several of our translation intermediates but the density in this area is much weaker in the unloaded state, suggesting that it is inactive (Extended Data Fig. 5a). The unloaded state matches the non-rotated *Pf*80S with E-site tRNA state observed in our single-particle cryo-EM structures; and while its precise function is still unclear, its prevalence in our in situ samples suggests some physiological relevance. It is possible that this represents an idle or storage ribosome state, although we do not observe density corresponding with any known ribosome-inactivating factors in our map^19^. Furthermore, the single tRNA is bound in the canonical E-site, excluding the possibility of a Z-site tRNA-associated inactive state^13,20^.

### CBQ-treated parasites contain fewer EF-bound *Pf*80S ribosomes

Next, we sought to gain insight into how treatment with a translation inhibitor might perturb malarial translation dynamics and inhibit protein synthesis. For this study, we decided to focus on CBQ, a leading antimalarial drug candidate currently in phase 2 clinical trials. CBQ has been established as a potent *Plasmodium*-specific translational inhibitor, active against multiple life-cycle stages of *Plasmodium* parasites^15^. While the mode of action of this promising antimalarial candidate is not known, recrudescent parasites exhibiting resistance to CBQ have been found to contain mutations in *Pf*eEF2 (refs. ^15,21^), strongly suggesting that *Pf*eEF2 is the molecular target. Molecular docking studies suggest that CBQ may bind to the region of *Pf*eEF2 that normally makes contact with the GTPase-binding site on the *Pf*80S, potentially blocking binding with the ribosome^21^.

**Fig. 4:**
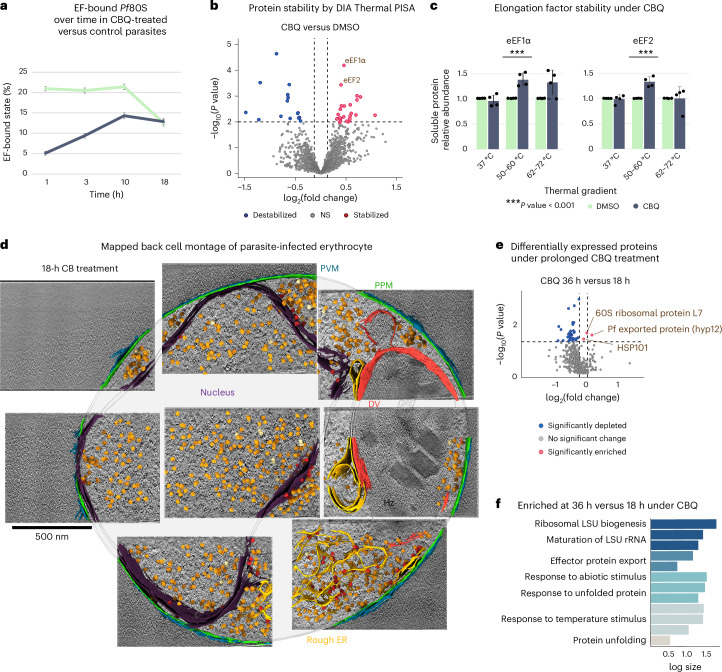
Molecular effects of CBQ treatment revealed by thermal PISA, integrated cell montages and quantitative proteomics. **a**, Graph of percentages of EF-bound states in CBQ-treated and DMSO control parasites, across four time points and four technical replicates. Percentages are based on total identified ribosomes after particle curation (*n* = 230,471). **b**, Volcano plot of differential abundance of soluble proteins in parasites treated with CBQ, relative to the DMSO vehicle control. Each protein is represented as a function of the log_2_ fold change (*x* axis) and −log_10_ *P* value (*y* axis) based on moderated *t*-test analysis carried out for each gradient independently. Hit selection cutoffs of *P* < 0.01 and a log_2_ fold change of >0.2 are represented by dashed lines. Drug-stabilized proteins, red; destabilized proteins, blue; proteins exhibiting no significant (NS) abundance change, gray. **c**, Relative soluble protein abundance of eEF2 and eEF1α identified in **a**. Protein abundance under CBQ treatment is plotted relative to the DMSO vehicle in each respective thermal gradient (*n* = 4 biological replicates). Significance changes (moderated *t*-test) are represented by asterisks (**P < 0.01 and ***P < 0.001). eEF2, *P* = 0.00037; eEF1α, *P* = 0.000064. Error bars represent the s.d. **d**, Integrated cell montage of a CBQ-treated parasite frozen, milled and imaged at 18 h after treatment. Segmented features are color-coded as indicated by corresponding colored labels. Mapped back ribosomes are colored as follows: orange, monomers; pale yellow, dimers and polysomes; red, membrane-bound ribosomes. **e**, Volcano plot of differentially expressed proteins in CBQ-treated parasites comparing 36 h to 18 h after treatment (52 versus 38 hpi). The mean average of each protein from three replicates (*n* = 3) is shown. The *y* axes show the log_10_ *P* value and *x* axes show the log_2_ fold change using a nested study design. Blue and red circles indicate proteins with significant change. Gray circles indicate proteins with no significant (*P* > 0.05) change in expression. Hit selection cutoffs of *P* < 0.05 and log_2_ fold change > 0.1 are represented by dashed lines. **f**, REVIGO-generated GO enrichment analysis of enriched proteins in CBQ-treated parasites at 36 h compared to 18 h. Bars are colored by REVIGO-designated cluster. A one-way ANOVA and pairwise comparison were used to compare each condition. The *y* axes represent the log_10_ number of annotated GO terms in *P.* *falciparum*. Each bar represents a subcluster.

Because of current limitations of the technique, it is not possible to visualize a small protein such as *Pf*eEF2 by itself within the cell. However, by comparing the relative abundance of different ribosomal states in untreated and CBQ-treated parasites, we can elucidate how CBQ alters malarial translation dynamics. Comparison of the percentage of *Pf*80S particles with occupied versus empty GTPase sites in CBQ-treated and control parasites across the four time points (Fig. 4a) revealed that the percentage of EF-bound *Pf*80S ribosomes decreased in CBQ-treated parasites when compared to control parasites through 10 h after treatment (30 hpi). Looking at the individual states (Extended Data Fig. 5b–d), we observe a slight drop in the percentage of eEF2-bound translocation state *Pf*80S and a marked decrease in the percentage of eEF1ɑ-bound decoding-1 and decoding-2 *Pf*80S states compared to untreated parasites.

To further elucidate the target space of CBQ, we established a robust ‘stability proteomics’ workflow combining cellular thermal shift (CETSA)^22,23^ with PISA^24^ and data-independent acquisition mass spectrometry (DIA-MS) to enable unbiased and untargeted identification of drug–target engagement events in live parasites. Briefly, parasites (37–41 hpi) were first treated for 1 h with CBQ (1 μM) or DMSO vehicle control. The parasites were subjected to thermal gradients of 50–60 °C or 62–72 °C, denaturing unstable protein subsets inside the cell (CETSA). Denatured protein subsets were then removed from the resulting parasite lysates and the remaining soluble proteins were quantified (PISA) with DIA-MS to maximize detection of low-abundance peptides. This workflow allowed us to detect drug-induced stabilizations across the entire parasite proteome in live parasites.

Differential abundance analysis (Fig. 4b,c) revealed significant CBQ-induced stabilization of both eEF2 (*P* = 0.00037, fold change = 1.32, moderated *t*-test) and eEF1ɑ (*P* = 0.000064, fold change = 1.37, moderated *t*-test) (Fig. 4b,c). Among 1,600 proteins quantified in the assay, CBQ-induced shifts in protein stability were observed for an additional 37 proteins at *P* < 0.01, including two stabilized aminoacyl-tRNA synthetases: aspartate and leucine (Extended Data Fig. 5e). Taken together, our data show depletion of eEF2-bound and eEF1ɑ-bound ribosomes and stabilization of both eEF2 and eEF1ɑ following CBQ treatment, which may be indicative of the mechanism by which CBQ inhibits malarial protein synthesis.

### Ribosome biogenesis is upregulated upon prolonged CBQ exposure

**Extended Data Fig. 6:**
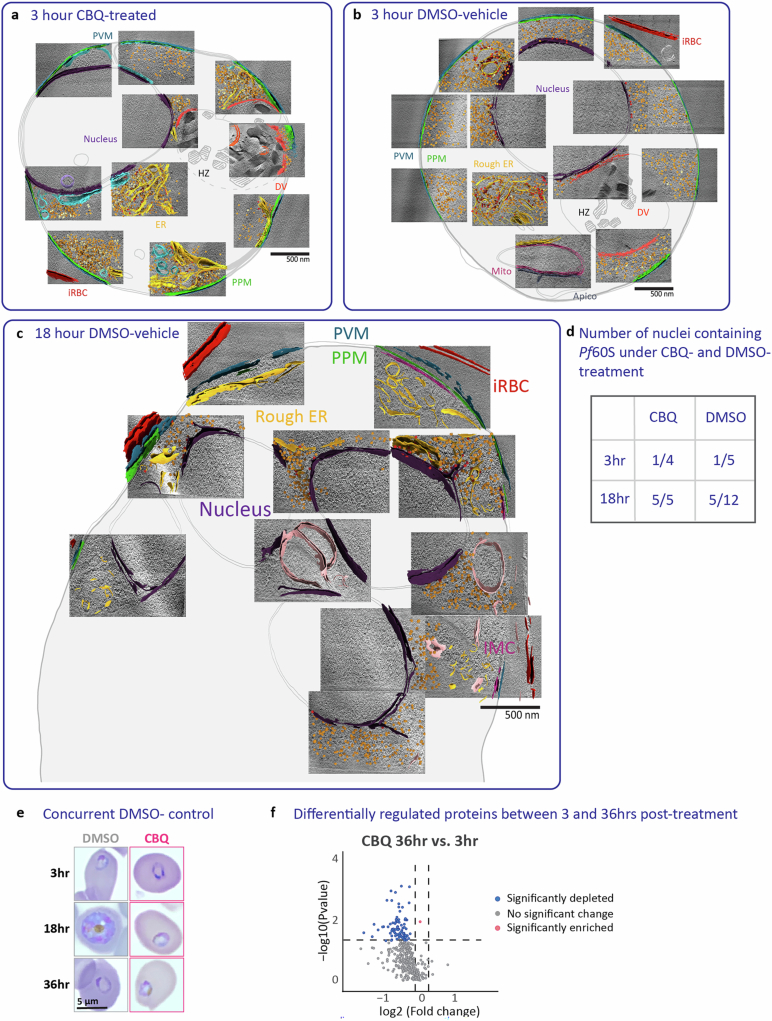
Details of integrated cell montages of CBQ- and DMSO-treated *P. falciparum* parasites and quantitative proteomic analysis. **a**, Integrated cell montage of a CBQ-treated parasite frozen, milled and imaged at 3 h post-treatment. Averaged central slices from tomograms collected on the cell are shown mapped onto a tracing of the cell, with ultrastructural segmentations and mapped back ribosome reconstructions overlaid on top. Segmented features are color coded as indicated by corresponding colored labels. Mapped back ribosomes are colored as follows: orange=monomers, pale-yellow=dimers and polysomes, red=membrane bound ribosomes. **b-c**, Integrated cell montage of a DMSO-vehicle parasite frozen, milled and imaged at 3 h (b) and 18 h (c) post-treatment. Segmentation is colored as in (a). **c**, Detailed view of the averaged central slices overlaid with segmentations containing the DV of the cell shown in (a). Segmented features are color coded as in (a). PVM, parasitophorous vacuolar membrane. PPM, parasite plasma membrane. EMS, endomembrane system. DV, digestive vacuole. Hz, hemozoin. ER, endoplasmic reticulum. **d**, Total count of number of nuclei containing Pf60S at 3 and 18 h under DMSO-vehicle and CBQ-treatment. **e**, Hema3-stained smears of parasites at 3, 18 and 36 h after addition of CBQ or DMSO- vehicle (23, 38, and 56 hpi). Parasites treated with DMSO-vehicle have egressed and reinvaded by the 36 hr (56 hpi) time point from three biological and technical replicates. **f**, Volcano plot showing differentially expressed proteins under CBQ-pressure comparing proteins present at 36 h to 3 h post treatment (56 to 23 hpi). The mean average of each protein from three replicates (n = 3) is shown. Y-axes show log10 p- value and x-axes show log_2_ fold change using nested study design. Dark coral and blue circles indicate proteins with significant enrichment and depletion, respectively. Grey circles indicate proteins with no significant (p > 0.05) change in expression. Hit selection cut-offs of p < 0.05 and log2 Fold-change of >0.1 are represented by dashed lines. One way ANOVA and pairwise comparison was used to compare each condition.

To gain a comprehensive understanding of the effect of CBQ inhibition of translation on the parasite, we integrated our local and global data by first mapping our STA *Pf*80S particles back into their originating tomograms (Fig. 4c and Extended Data Fig. 6a–c). Next, to gain the full cellular context, we then mapped averaged central slices from tomograms collected on each cell back onto a lower-magnification image of the cell, then overlaid ultrastructural segmentations and mapped back ribosome reconstructions on top, yielding an integrated cell montage spanning multiple length scales (Fig. 4c and Extended Data Fig. 6a–c). From these cell montages, we see that, on the ultrastructural level, CBQ-treated parasites remain constant in size and organellar organization across all time points, while DMSO control parasites increase dramatically in size and organellar content. Strikingly, the nuclei in our CBQ-treated parasites contain an unusual abundance of ribosome-like particles in the nucleus at 18 h after treatment (38 hpi) (Fig. 4c). We hypothesized that these could be preassembly 60S particles accumulating in the nucleus, perhaps resulting from a delayed attempt by the parasite to increase ribosome biogenesis. Indeed, upregulation of ribosome biogenesis in response to inhibition of protein synthesis was previously reported in other organisms^25^.

While we were unable to obtain enough of these nuclear ribosome-like particles to yield a separate STA reconstruction, we performed quantitative proteomics on drug-treated early-trophozoite-stage (20 hpi) parasites isolated at 3, 18 and 36 h after treatment (23, 38 and 56 hpi). In this time frame, DMSO-treated parasites progressed normally through the schizont stage, replicated, egressed and then reinvaded by ~28 h after treatment (~48 hpi); their proteomes cycled through three distinct blocs of gene expression profiles^26^ (Extended Data Figs. 3 and 6e), making them an unsuitable basis for comparison. Consequently, we used the proteome of the 3 h CBQ-treated parasites as a baseline for determining protein enrichment or turnover at the 18-h and 36-h time points. We observed enrichment of a small number of proteins in CBQ-treated parasites across both the 3–18-h and 18–36-h intervals, despite an overall depletion of proteins (Fig. 4d and Extended Data Fig. 6f). Gene ontology (GO) enrichment analysis on these differentially regulated proteins in PlasmoDB^27^ revealed an enrichment of proteins involved in ribosome biogenesis and ribosomal RNA (rRNA) maturation between 18 and 36 h (Fig. 4e), supporting our hypothesis that ribosome biogenesis is upregulated in parasites following prolonged CBQ exposure.

## Discussion

In this study, we reveal the molecular details of the native malarial translation elongation cycle and perturbations in the translation dynamics, proteome and ultrastructure of malaria parasites after treatment with a *Plasmodium*-specific translation inhibitor. Our in situ cryo-ET reconstructions reveal an ensemble of translation intermediates sampled by the native Pf80S ribosome, deepening our understanding of translation elongation in eukaryotic pathogens.

Despite added challenges stemming from the unique biology of the parasite, the malarial translation elongation cycle revealed here is more complete, containing five active translation intermediates with the E-site occupied (the POST, non-rotated PRE, rotated-1 PRE, rotated-2 PRE and unloaded states) that have not been described within eukaryotic cells. This may be a consequence of nuanced differences in *P.* *falciparum* translation dynamics compared to those of model eukaryotes^28,29^, underscoring the importance of studying translation in its physiological context to capture dynamic conformational heterogeneity that may be lost in in vitro studies.

Our work highlights the power of in situ cryo-ET for phenotypic characterization across multiple length scales, simultaneously capturing changes in a protein’s distribution between functional states, its interactions with binding partners and its subcellular localization in response to perturbation. At the molecular level, comparing ribosomal states in untreated and CBQ-treated parasites revealed a marked depletion of EF-bound ribosomes in drug-treated samples, suggesting that CBQ disrupts normal ribosome function by interfering with translation elongation. Further supporting this hypothesis, we observed stabilization of *Pf*eEF2 and *Pf*eEF1α upon CBQ treatment by thermal PISA. On the level of subcellular localization, we visualized accumulation of ribosome-like particles within the nuclei of CBQ-treated parasites, suggesting an increase in ribosome biogenesis following prolonged translation inhibition, which was further supported by our proteomic analysis. By integrating molecular, ultrastructural and cellular readouts, this approach represents a major advance in the resolution of observable phenotypic changes, bridging the resolution gap between structural and cellular biology and providing unprecedented insight into the consequences of translation inhibition in *Plasmodium* parasites.

Beyond malaria, our study demonstrates the broader potential of in situ cryo-ET for uncovering molecular mechanisms in infectious disease research. By directly visualizing drug-induced perturbations within the native cellular milieu, we provide new avenues for mechanistic investigations of host–pathogen interactions and therapeutic interventions at unprecedented resolution.

## Methods

### Parasite culture and synchronization

We used NF54attb:EXP2-mNeonGreen *P.* *falciparum* parasites^30^ for all in situ cryo-ET studies, providing us with a fluorescent signal with which to locate promising areas containing parasite-infected cells on our vitrified cryo-ET grids in the Leica cryo-CLEM instrument. For consistency, we used the same parasite line for all other experiments unless otherwise stated. For all experiments, unless otherwise specified, asexual NF54attb::EXP2-mNeonGreen *P.* *falciparum* parasites were cultured following a protocol adapted from Duffy et al.^31^. Parasites were cultured in O+ or A+ erythrocytes (New York Blood Center), with continuous gentle shaking, at 37 °C under 5% O_2_, 5% CO_2_ and 90% N_2_ in complete RPMI (cRPMI): RPMI 1640 medium (Sigma, R6504-50L) supplemented with 0.05 mg ml^−1^ hypoxanthine (Sigma, H9377-100G), 0.06 mg ml^−1^ sodium hydroxide (Fisher Scientific, SS255-1), 0.8 mg l^−1^ thymidine (Sigma, T1895-1G), 0.04 mg ml^−1^ sodium pyruvate (Sigma, P5280-256), 2.25 mg ml^−1^ sodium bicarbonate (Sigma, S6014-500G), 5.9 mg ml^−1^ HEPES (Sigma, H4034-500G), 0.67 mg ml^−1^ glucose (Sigma,G7021-1KG), 0.01 mg ml^−1^ gentamycin (Sigma, G1271-100ml) and 0.5% Albumax II (Gibco, 11021-045), at a hematocrit of 2% at <10% parasitemia. Unless otherwise specified, reagents were purchased from Sigma.

For parasite synchronization, unless otherwise stated, parasites^30^ were repeatedly synchronized first using a cushion of 65% Percoll (Cytiva, 17089109) and then allowed to reinvade for 4–6 h before further synchronizing to the ring stage with 5% sorbitol (Fisher Scientific, AA3640436; Sigma, 1077581000) and placed back into culture to progress to the desired stage for each experiment.

### Hema3-stained 48-h drug treatment time course

Synchronized parasites at the early trophozoite stage (20 hpi) were pelleted at 800*g* for 3 min at room temperature and then enriched by gelatin flotation in 0.7% gelatin at 37 °C for 1 h. The trophozoite-containing supernatant was collected, washed and resuspended in 1 ml of RPMI with 20 nM CBQ or 2 pM DMSO at 5% hematocrit into a 24-well plate (Corning, 353047). A small aliquot of culture was collected every 4 h over the next 48 h, smeared, stained using Hema3 fixative and solutions (Fisher Healthcare, 122-911) and *n* = 25–81 cells were imaged at ×100 magnification on an Echo Revolve R4 microscope (Extended Data Fig. 3).

### Single-particle cryo-EM

#### Ribosome purification for single-particle cryo-EM

Synchronized trophozoite stage parasites were harvested with saponin (0.0125%) in 1× PBS with Roche cOmplete protease inhibitor tablets (Roche) and washed with 1× PBS with protease inhibitor tablets. The dry pellet was flash-frozen and stored at −80 °C. Frozen parasite pellets were thawed and resuspended in polysome lysis buffer (20 mM HEPES pH 7.5, 150 mM NaCl, 25 mM MgCl_2_, 1% Triton X-100, 0.1 mg ml^−1^ cycloheximide and protease inhibitor tablets) and 80S ribosomes were purified over a 10–50% sucrose gradient made in the same buffer.

#### Single-particle sample freezing and data collection

Purified ribosomes were incubated with 50 μM CBQ for 30 min at room temperature and applied to glow-discharged R2/2, 200-mesh Au with ultrathin carbon film Quantifoil EM grids (Quantifoil Micro Tools) and vitrified in liquid ethane using an FEI Vitrobot Mark IV. Grids were screened on a Glacios transmission EM (TEM) instrument at 200 kV with Falcon 3EC direct electron detector. High-resolution data were collected on an FEI Titan Krios at 300 kV with a Gatan K3 camera and quantum energy filter (Gatan K3-BioQuantum). A total of 17,190 videos were acquired at a magnification of ×105,000 (0.83 Å per pixel).

#### Single-particle data processing, modeling and analysis

The cryo-EM data-processing workflow for the single-particle *Pf*80S data is shown in Extended Data Fig. 2. After initial preprocessing in RELION^32^, a manually pretrained model in crYOLO^33^ was used for particle picking. In total, 1,873,509 particles were extracted from 17,190 micrographs and initially binned by 4 for one round of two-dimensional (2D) classification in RELION. After filtering out low-quality particles, a total of 1,410,615 particles were aligned with 3D refinement in RELION using a cryoSPARC^34^ ab initio map as reference. All subsequent image-processing steps were performed using RELION. To separate rotated (rtPf80S) and non-rotated (nrtPf80S) states, particles were re-extracted at bin 2 and focused classification of the 40S body was performed. Different tRNA states were identified using rounds of focused classification on individual translation intermediates. Maps were then unbinned to the full pixel size of 0.83 Å per pixel and refined and half-maps were postprocessed in RELION to yield final maps. Local resolution estimation was also performed in RELION.

#### Single-particle atomic model building

Protein Data Bank (PDB) 8TPU generated for the STA consensus model was used as a basis for all our new models, as described later. PDB 8TPU was rigid-body fitted into the different maps of the three non-rotated states using ChimeraX^35^. For the rt-E and rt-PE, the 40S and 60S subunits of PDB 8TPU were separately rigid-body fitted into the maps to account for the rotation of the 40S. Chains of tRNAs and mRNA of other models were used to get an initial spatial placement and structure: A-site tRNA (PDB 3J0O, chain Y), P-site tRNA (PDB 3JBN, chain S9), E-site tRNA in rotated states (PDB 3JBO, chain 7) and mRNA (PDB 5LZS, chain hh). The selected chains were then rigid-body fitted into the corresponding single-article analysis maps in ChimeraX for a coarse placement. Afterward, the fitted components of each new model (60S, 40S, tRNAs and mRNA) were merged in Coot^36^, creating five new models. Subsequently, manual adjustments and local real-space refinement was conducted in Coot for each of the models to fit the tRNAs, mRNA and parts of the 40S to the individual maps. If density was visible in the peptide exit tunnel, building of the nascent chain as a poly(A) stretch was also conducted in Coot. All adjustments in Coot were followed by refinement and subsequent validation of the new model using the PHENIX software suite^37^ (Extended Data Table 1). Manual adjustment and refinement were performed in an iterative process to optimize the model. Final models were deposited to the PDB under accession codes 9BUP, 9BUQ, 9BUS, 9BUT and 9BUU (Extended Data Table 1).

### Cryo-ET

#### Crude fractionation of ring-stage parasites (ex vivo)

Parasites from the wild-type D10 line^38^ were synchronized by 0.5 M alanine and 10 mM HEPES and isolated at the late ring stage to the early trophozoite stage (20 hpi) at 8–15% parasitemia by pelleting and then resuspending in AIM buffer (KCl 120 mM, NaCl 20 mM, glucose 20 mM, HEPES 6 mM, MOPS 6 mM, MgCl_2_ 1 mM and EGTA 0.1 mM, pH 7.0) and an equal volume of AIM buffer supplemented with 0.1% saponin. After washing the pellet three times with AIM buffer, the pellet was resuspended in MESH buffer (mannitol 225 mM, sucrose 75 mM, MgCl_2_ 4.3 mM, Tris 10 mM, EGTA 0.25 mM and HEPES 15 mM, pH 7.6) supplemented with 1 mM PMSF and 1 µl ml^−1^ fungal protease inhibitor cocktail. Parasites were slowly disrupted by N_2_ cavitation (4639 Cell Disruption Bomb, Parr) at 1,000 psi at 4 °C and then spun down at 900*g* for 5 min to separate solid from soluble fraction. The solid phase containing nucleus and large debris was removed. The cloudy supernatant was passed through a magnetic column (Miltenyi Biotec) to remove hemozoin. The elute was pelleted by centrifugation at 20,000*g* for 30 min at 4 °C. Pelleted fractions were flash-frozen in liquid nitrogen and stored at −80 °C. Samples were thawed and exchanged into 20 mM HEPES pH 7.5, 150 mM NaCl and 5 mM MgCl_2_ directly before freezing grids for ex vivo cryo-ET.

#### Parasite enrichment and preparation for in situ studies

To enrich merozoites, parasites synchronized as described above were allowed to progress to the schizont stage (42–44 hpi), purified using a 65% Percoll (Cytiva) cushion and then treated with 10 µM E-64 (Sigma, E3132) for 4–8 h until mature merozoites formed. Merozoites were then mechanically released from the host erythrocyte by passage through a 1.2-µm syringe filter (Pall Life Sciences, 4656), pelleted at 2,000*g* for 5 min.

To enrich trophozoites, parasites synchronized as described above were allowed to progress to the trophozoite stage (20 hpi). Drug-treated trophozoites were treated with the appropriate drug prepared in cRPMI or with cRPMI containing DMSO vehicle control. The drug used esd 20 nM CBQ (ApexBio, A8711) prepared in DMSO. iRBCs were isolated after 1, 3, 10 or 18 h of treatment. For each time point, an aliquot of purified trophozoites were placed back into culture to monitor parasite development over the following 48 h. Blood smears of all parasites were stained using Hema3 fixative and solutions (Fisher Healthcare) and imaged at ×100 magnification using an Echo Revolve R4 microscope at each time point.

#### Sample freezing and cryo-CLEM

For in situ cryo-ET, 3.5 µl of iRBCs in cRPMI were applied to glow-discharged R2/2, 200-mesh Au, Cu, carbon or SiO_2_ Quantifoil EM grids, followed by manual back-blotting with Whatman No. 1 filter paper and plunge-freezing in liquid ethane using a custom-built manual plunger. Frozen grids were clipped into AutoGrids (Thermo Scientific) and assessed for ice thickness and cell density using a cryo-CLEM instrument (Leica Microsystems). For ex vivo cryo-ET, crudely fractionated lysate from untreated ring-stage parasites were plunge-frozen in liquid ethane in a Vitrobot Mark IV semiautomated plunge freezer (Thermo Scientific) on glow-discharged R2/2, 200-mesh Au and carbon Quantifoil EM grids (Quantifoil Micro Tools).

#### FIB-SEM

Grids were loaded into an Aquilos cryo-FIB-SEM instrument (Thermo Scientific) and lamellae were created as previously described^6^. In brief, eucentric height was determined for suitable target sites, after which the grid was sputter-coated with platinum metal and then coated with trimethyl(methylcyclopentadienyl)platinum(IV) using the onboard gas injection system. Tension release trenches were milled at an angle of 40°, after which the milling process was performed at angles of 7–8° at 30 kV, using currents of 0.5 nA, 0.3 nA., 0.1 nA, 50 pA and 10 pA for the FIB. Final lamellae were polished to a thickness of 150–200 nm.

#### Data collection

Data collection was performed on three high-end cryo-TEM instruments operating at 300 kV: two Titan Krios (Thermo Fisher) with K3 cameras and Gatan imaging filter systems (Gatan) and one Titan Krios with Falcon IV (Thermo Fisher) and Selectris energy filter (Thermo Fisher) (Extended Data Table 2). All data were collected in .mrc format in super-resolution mode at a magnification of ×42,000 (physical pixel size of 2.094 Å per pixel) or ×53,000 (physical pixel size of, 1.66, 1.699 or 1.6 Å per pixel), depending on the microscope (Extended Data Table 2). SerialEM (for K3 camera)^39^ or EPU Tomo (for Falcon IV camera) (Thermo Scientific) acquisition software was used to set up and collect tilt series with a total dose of 120 *e*− per Å^2^, over 41 tilts comprising ten frames per tilt. Each tilt series was collected over a range of 120° in 3° increments following a dose-symmetric scheme, starting at a pretilt angle defined by the milling angle (7–8°).

#### Cryo-ET data processing and analysis

Collected tilt series (Extended Data Table 2) were preprocessed using Warp (version 1.0.9)^40^ including 2D contrast transfer function correction, motion correction and creation of tilt stacks. Tomogram alignment and reconstruction were performed without pretilt correction in AreTomo^8^ and denoised using Topaz 3D denoise^10^.

##### STA

The crYOLO semiautomated particle picker^33^ was manually trained on denoised tomograms from each dataset and the resulting model was applied to all denoised tomograms of the same dataset (Extended Data Table 2). The resulting particle picks were manually curated in EMAN2 e2spt_boxer.py^41^ to exclude false positives. Subtomograms were extracted in Warp (version 1.0.9) using alignment files from AreTomo and averaged and refined in RELION3.1 (ref. ^32^). Resulting reconstructions and particle orientations were imported into M (version 1.0.9)^7^ to sequentially refine particle pose, image and volume warp and defocus parameters (Extended Data Table 3). Improved particles were re-extracted and again refined in RELION. This scheme was continued until the consensus refinement reached the Nyquist limit of 4.1 Å and was used for 3D focused classifications (Extended Data Fig. 4a).

##### Focused 3D classification

To separate the individual translation intermediates, several rounds of focused classification were performed on all particles in the refined consensus map at 2.094 Å per pixel (Extended Data Fig. 4a). Classifications were performed in three steps. A first classification was performed to separate particles with and without density in the GTPase site using a mask around the GTPase site (EF mask). In a second step, particles with and without GTPase site density were both separately subjected to another round of classification, separating out the various EF-bound or unclear GTPase site density states (eEF1α and eEF2) for particles containing density in the GTPase site or separating out particles on the basis of occupancy of the A-site or P-site tRNA for particles with no density in the GTPase site. In some cases, a mask around the A-site tRNA (A-tRNA mask) was used to separate out the different EFs. In a third step, a mask around the P*-site and partial E-site is used to separate out P*-site and E-site occupancies (P*E mask). Sometimes a fourth classification was necessary using the abovementioned masks or a mask covering the GTPase site, A-site, P*-site and E-site (full mask) to get final classes. All classifications were repeated at least twice with similar results. A full mask of the GTPase-binding and tRNA-binding sites was generated in Xmipp^42^ on the basis of the refined consensus map. The GTPase site, A-site and P*E-site masks were generated from the full mask by deleting unwanted regions using the volume-erase function in ChimeraX. Soft masks were then created from these in RELION3.1_mask_create, using a 3-pixel binary mask extension and 4-pixel soft edge.

##### Assigning STA Pf80S states on the basis of comparison to previously published structures

As illustrated in Fig. 3, our datasets contain intermediates making up the translocation, decoding and peptidyl-transfer phases of elongation, including the following states: decoding-1 (eEF1α, A/T-site, P*-site and E-site)^43^ (Fig. 3a), decoding-2 (eEF1α, A/T-site and P*-site)^44^ (Fig. 3b), non-rotated pretranslocation (A-site, P*-site and E-site)^43^ (Fig. 3c), rotated-1 pretranslocation backrolled (A/P-site and P*-site)^13,45^ (Fig. 3d), rotated-1 pretranslocation (A/P-site and E-site)^46^ (Fig. 3e), rotated-2 pretranslocation (P-site and E-site)^46^ (Fig. 3f), translocation (eEF2, P*-site and E-site)^43,47^ (Fig. 3g) and post-translocation (P*-site and E-site)^43^ (Fig. 3h). Of these, the post-translocation and rotated-2 pretranslocation states have been characterized in previous *P.* *falciparum* single-particle cryo-EM studies^17,48^. Our decoding-1 and decoding-2 states both contain an eEF1α A/T-site tRNA and P-site tRNA bound in a similar fashion to published decoding structures of rabbit eEF1α A-site-bound 80S ribosomes or bacterial EF Tu-bound 70S ribosomes, with the decoding-1 state also containing an E-site tRNA^46^ (Fig. 3a). Our decoding-2 state is consistent with the eEF1α, A/T-site and P-site class found in the protozoan *Dictyostelium discoideum*^12^ (Fig. 3b) and an AlphaFold2 model of *Pf*eEF1α (*Pf*eEF1α _AF_, AF-QI80P6) fits well into the density at the GTPase-binding site in both decoding state reconstructions. Our non-rotated pretranslocation (corresponding to the classical iPRE state described in previous studies), rotated-1 pretranslocation backrolled, rotated-1 pretranslocation, rotated-2 pretranslocation, translocation and post-translocation states (Fig. 3c–h) all match previously published human and *P.* *falciparum* 80S single-particle cryo-EM structures^13,17,46,47,49^. Our translocation state map contains a *Pf*eEF2 bound in an extended conformation as seen in published human or yeast ribosome-bound eEF2 structures and confirmed by fitting in a model of *Pf*eEF2 generated using SWISS-MODEL (Fig. 3g)^47,50–55^.

##### Calculating distribution of particles with occupied versus empty GTPase sites by condition

All particles in each final classified state were refined in RELION3.1 to yield a final consensus reconstruction of each state. Details of the reconstructions of the individual states can be found in Extended Data Table 4. To determine the percentage of EF-bound states in CBQ and CTL particles, we took the final RELION Refine3D star file for each EF-bound state (including unassigned states with and without GTPase) and wrote out the number of particles for each condition and time point indicated (Fig. 3j). Combining the number of particles of the same data point yielded the exact number of particles per condition per time point. From this new combined number, we were then able to calculate the percentage of EF-bound ribosomes per condition, per time point, as a percentage of the total number of ribosomes in each dataset.

##### Segmentation

Segmentations were generated in a semiautomated manner using EMAN2 (ref. ^41^) and Membrain-seg^56^. Briefly, models were trained on manual membrane annotations for each tomogram, applied to the tomogram and then manually separated and colored for display and figure making in UCSF ChimeraX^35^.

#### STA atomic model building

Models for *Pf*eEF1α_AF_ and *Pf*eEF2_AF_ were downloaded from the AlphaFold2 (ref. ^57^) website under accession codes AF-QI80P6 (*Pf*eEF1α_AF_) and AF-Q9NDT2-F1 (*Pf*eEF2_AF_). SWISS-MODEL^51–55^ was used to generate alternative models for *Pf*eEF1α and *Pf*eEF2 based on human eEF1α (PDB 8G6J) and human eEF2 (PDB 6Z6M). To assess translational states and confirm binding positions, previously published models or maps of full 80S ribosomes were fitted into our *Pf*80S maps. PDB 3JBP was used as a basis for our new model. The model was rigid-body fitted into the highest-resolution consensus structure using ChimeraX. Manual adjustments to the model were made in Coot^36^, followed by refinement and subsequent validation of the new model using the PHENIX software suite^37^ (Extended Data Table 4). Manual adjustment and refinement were performed iteratively to optimize the model. The refinement strategy included minimization_global, local_grid_search, occupancy, adp and hqh_flips for 100 iterations and five macro cycles using secondary-structure and noncrystallographic symmetry constraints or default settings. The model was submitted to the PDB under accession code 8TPU.

#### Computational polysome analysis

Polysomes were computationally identified using the relative distances between the entry and exit sites of all ribosomes in a tomogram. First, exit and entry sites were defined as *x*, *y* and *z* coordinates in the consensus reconstruction, relative to the point of origin in ChimeraX. Then, the maximum allowed distance between exit of the leading and entry of the following ribosome in a *P.* *falciparum* polysome was defined by simulating the placement of two ribosomes in a polysome on the basis of low-resolution densities for neighboring ribosomes in our reconstructions and confirmed with previously performed polysome analysis^44^. The measured distance in the simulated polysome was 138 Å; on this basis, a threshold of 150 Å was set. The determined exit and entry site coordinates were then applied to the refined rotation angles and center-of-mass shifts for each particle, enabling calculation of the specific positions of the entry and exit sites of all individual ribosomes comprising the consensus reconstruction. Using the newly generated coordinates, we identified the leading and following ribosome for each ribosome pair (*i*, *j*) by recording the distance between ribosome *i*’s entry from ribosome *j*’s exit at matrix index (*i*, *j*) and vice versa at matrix index (*j*, *i*). Between these two matrix indices, only the smaller index (shorter distance) was considered and the larger one was replaced with an arbitrarily large number. Next, we applied the defined distance threshold of 150 Å to this filtered matrix, setting all values above this threshold to 0. The nonzero entries of the new matrix were used to add ribosomes to disjoint sets, with each set representing a monosome (one element), a dimer (two elements) or a polysomes (more than two elements).

#### Mapping back of ribosomes

The relionsubtomo2ChimeraX Python script^58^ was adapted to include polysome information. The script produces a .cxc file, containing information on ribosome poses in the original tomogram based on RELION star file input, and enables positioning of reconstructions in the tomographic volume by ChimeraX. On the basis of results obtained from the polysome analysis described above, the code assigns different colors to monomers (orange), dimers and polychains (butter yellow).

#### DIA thermal PISA

The experiment was carried out in four biological replicates. Synchronized *P.* *falciparum* 3D7 mature-stage parasites (37–41 hpi) were exposed to 1 µM concentration of CBQ (Sapphire Bioscience) or the DMSO (Sigma) vehicle control for 1 h in standard culture conditions. Parasites were pelleted through centrifugation, resuspended in DPBS (Gibco) supplemented with 1 µM CBQ and DMSO and split into 13 identical aliquots each. Aliquots were transferred onto a 96-well plate and heated in a PCR machine (Bio-Rad) for 3.5 min to different temperatures across a 50–72 °C gradient (at 2 °C intervals), followed by 3 min at 4 °C. Following addition of the same volume of DBPS with 0.8% IGEPAL CA-630 (Sigma), cells were lysed by three cycles of flash-freezing and thawing using liquid N_2_, followed by 10× mechanical sheering with a 29-gauge needle-syringe and denatured protein removal through filtration at the 0.2 µM level. The soluble phase was recovered and pulled together in equivolume ratios into two samples: gradient 1, 50–60 °C; gradient 2, 62–72 °C. Sample preparation for proteomic analysis was carried out using a modified SP4 protocol^59^. In brief, 20 µg of protein was reduced (20 mM TCEP and 100 mM TEAB) for 10 min at 95 °C and alkylated with 55 mM chloroacetamide for 30 min. Following the addition of 20 µl of PureCube Carboxy magnetic beads (Cube Biotech) and neat ice-cold acetonitrile to a final concentration of 80%, samples were incubated on a thermomixer for 20 min at room temperature at 800 rpm and pelleted down at 3,000*g* for 5 min. Beads were washed three times with 80% ethanol and, following SN removal, dried in a SpeedVac. Dried beads were resuspended in 100 mM TEAB and subjected to sequential digestion with LysC (3 h, 1:50 enzyme-to-protein ratio) and trypsin (overnight, 1:50 enzyme-to-protein ratio). The resulting digest was acidified with 10% TFA to a 1% final concentration and desalted on T3 C18 stage tips (Affinisep) according to the manufacturer’s instructions.

#### MS data acquisition and data analysis

Following resuspension in 0.1% formic acid and 2% acetonitrile, peptide samples were loaded on to a C18 fused silica column (inner diameter, 75 µm; outer diameter, 360 µm; length, 15 cm; 1.6-µm C18 beads) packed into an emitter tip (IonOptics) separated on a 45-min analytical gradient on a Neo Vanquish liquid chromatography system (Thermo Scientific) interfaced with MS (Orbitrap Eclipse Tribrid MS instrument, Thermo Scientific) and analyzed in DIA mode. Peptide identification was carried out in DIA-NN (version 1.8.1) using an in silico spectral library generated from UniProt *P.* *falciparum* (UP000001450) and human (UP000005640) reference proteomes. One missed cleavage and two variable modifications (ox(M) and Ac(N-term)) were allowed. Differential abundance data analysis (moderated *t*-test^60^) of *P.* *falciparum* proteins was conducted in the R environment using precursor-normalized MaxLFQ data for proteins detected with ≥2 peptides. Hit selection criteria included a *P* value < 0.01 and log_2_ fold change > 0.2 in protein abundance and protein detection across all samples in the comparison.

#### Proteomics

Synchronized parasites at 2% hematocrit and <10% parasitemia were expanded to 1.3 L, then treated with 20 nM CBQ in cRPMI (at 20 hpi) and incubated at 37 °C. Treated parasites were harvested by washing twice with 1× PBS, flash-frozen and stored at −80 °C. Tandem-mass-tag-based quantitative proteomics was performed at the Weill Cornell Medicine Proteomics and Metabolomics Core Facility. For all experiments, cells were grown and treated in biological triplicates. CBQ-treated pellets were isolated at 3 h, 18 h and 36 h after treatment (23, 38 and 56 hpi). A concurrently cultured DMSO-treated sample was smeared at 3 h, 18 h and 36 h after treatment (23, 38 and 8 hpi (in another cycle)) to confirm normal parasite development.

Pellets were provided to the facility where proteins were extracted, reduced, alkylated and digested with trypsin. Desalted peptides (50 µg) were labeled by 18-plex TMTpro (Thermo Fisher Scientific), of which a small aliquot was mixed and analyzed by liquid chromatography (LC)–MS to determine labeling efficiency and necessary ratio mixing. All samples were mixed at equal ratios and fractionated by offline reverse-phase LC into 12 fractions. Then, 5% of each fraction was run by LC–MS for global proteomics and the remaining 95% was enriched for phosphopeptides using TiO_2_ beads (GL Sciences). An EASY-nLC 1200 coupled online to a Fusion Lumos MS instrument (Thermo Fisher Scientific) was used for LC–MS. Buffer A (0.1% formic acid in water) and buffer B (0.1% formic acid in 80% acetonitrile) were used as mobile phases for gradient separation. A 75-µm × 15-cm chromatography column (ReproSil-Pur C18-AQ, 3 µm; Dr. Maisch) was packed in house for peptide separation. Peptides were separated with a gradient of 10–40% buffer B over 110 min and 40–80% B over 10 min at a flow rate of 300 nl min^−1^. The Fusion Lumos MS instrument was operated in data-dependent mode. Full MS scans were acquired in the Orbitrap mass analyzer over a range of 400–1,500 *m*/*z* with a resolution of 60,000 at 200 *m*/*z*. The top 15 most abundant precursors with charge states between 2 and 6 were selected with an isolation window of 0.7 Th by the quadrupole and fragmented by higher-energy collisional dissociation with normalized collision energy of 40. MS/MS scans were acquired in the Orbitrap mass analyzer with a resolution of 30,000 at 200 *m*/*z*. The automatic gain control target value was 1 × 10^6^ for full scans and 5 × 10^4^ for MS/MS scans; the maximum ion injection time was 100 ms for MS scans and 54 ms for MS/MS scans.

Data analysis was performed at the Center for Proteome Analysis at the Indiana University School of Medicine using Proteome Discoverer 2.5 (Thermo Fisher Scientific). The raw data were searched against *Homo sapiens* protein (downloaded May 13, 2022; 20,292 sequences), *P.* *falciparum* 3D7 protein (downloaded June 4, 2021; 5,381 sequences) and common laboratory contaminant (73 sequences) databases using Sequest HT. Full trypsin digestion with a maximum of three missed cleavages, a precursor mass tolerance of 10 ppm and a fragment mass tolerance of 0.02 Da was applied. Static modification of carbamidomethyl C was used. Dynamic peptide modifications were set at a maximum of three per peptide including oxidation of M, phosphorylation of S, T and Y, deamidation of N and Q, TMTpro of K and peptide N terminus and acetyl-M loss or M loss plus acetyl on protein N terminus. Percolator false discovery rate cutoffs of 0.01% strict and 0.05% relaxed at the peptide spectrum match level were used. The IMP-ptmRS node was used for phosphosite localization confidence scoring. At the consensus level, unique and razor peptides were used for quantification with reporter abundance based on S/N, isobaric quantification corrections applied, a coisolation cutoff of 30% and an S/N threshold cutoff of 6. Normalization was performed using total peptide amount with no scaling. Results were filtered for *P.* *falciparum* proteins and phosphopeptides and exported to Excel (Microsoft). Raw and processed MS data were uploaded to the MassIVE repository with accession number MSV00009987.

The relative quantitation of protein and phosphopeptide intensities was log-transformed and normalized using the median intensity value per sample. A one-way analysis of variance (ANOVA) and pairwise comparison were used to compare each condition. Volcano plots showing differential expression of proteins were generated using ggplot in R Studio^60^. GO analysis of differentially expressed proteins was performed with the GO enrichment tool in PlasmoDB focused on biological processes with a *P*-value cutoff of 0.05. Generated GO terms were imported into the REVIGO^61^ (reduce and visualize GO) web server and analyzed for clustered visualization.

#### Figures and plots

All figures were generated in Adobe Illustrator and graphs were plotted in Microsoft Excel or R studio. ChimeraX was used to visualize STA reconstructions and segmentations. Central slice images were generated in IMOD^62^. Lamella montages were compiled in Adobe Photoshop.

### Reporting summary

Further information on research design is available in the Nature Portfolio Reporting Summary linked to this article.

## Online content

Any methods, additional references, Nature Portfolio reporting summaries, source data, extended data, supplementary information, acknowledgements, peer review information; details of author contributions and competing interests; and statements of data and code availability are available at 10.1038/s41594-025-01632-3.

## Supplementary information

Reporting Summary

## Source data

Source Data Table 1 MS hitlist for DIA thermal PISA.

Source Data Table 2 MS summary for proteomics.

## Figures and Tables

**Extended Data Table 1:**
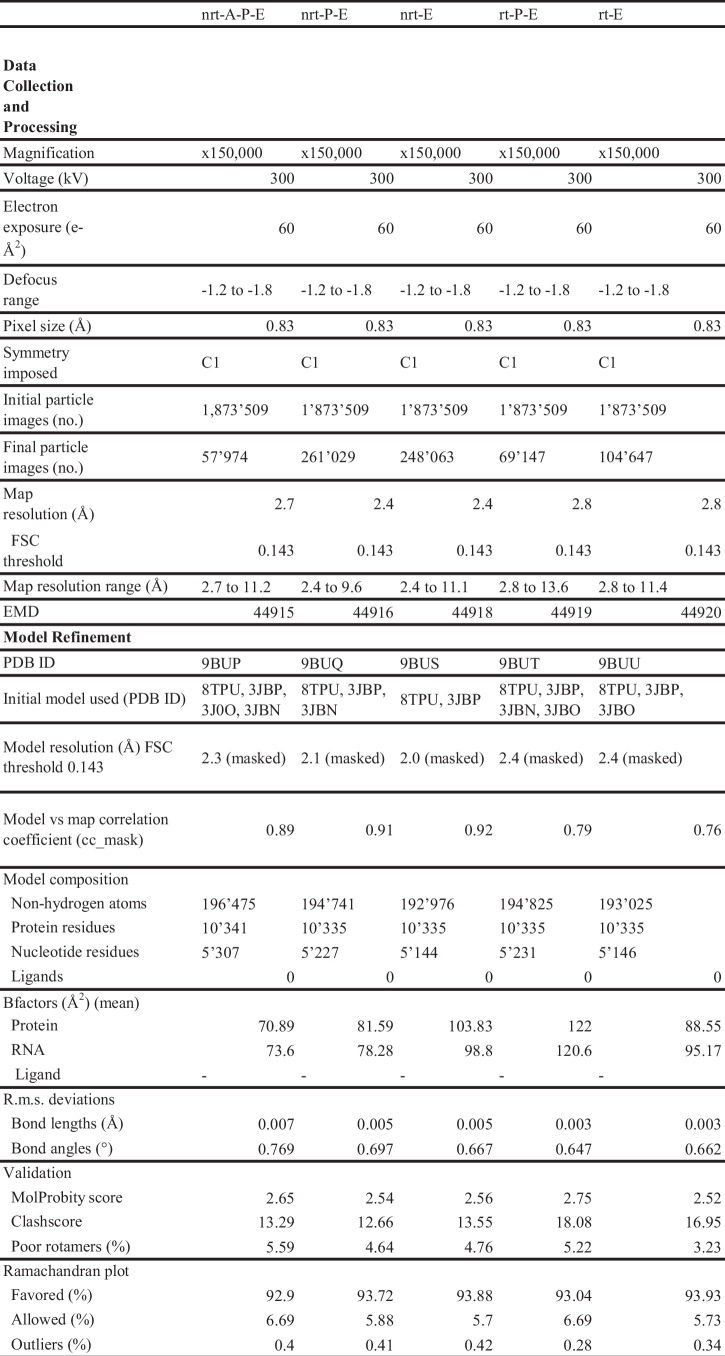
Data Collection, processing and Model building and refinement for SPA

**Extended Data Table 2:**
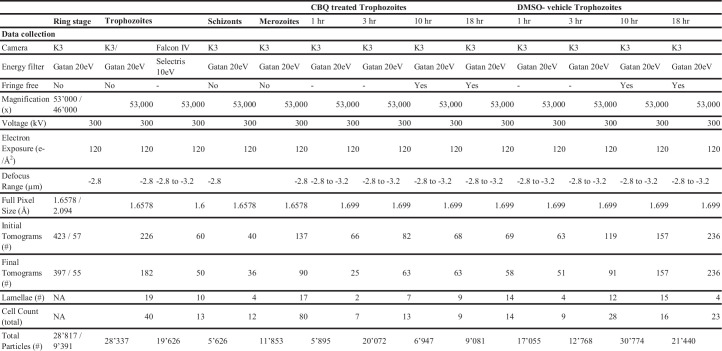
STA Data Collection

**Extended Data Table 3:**
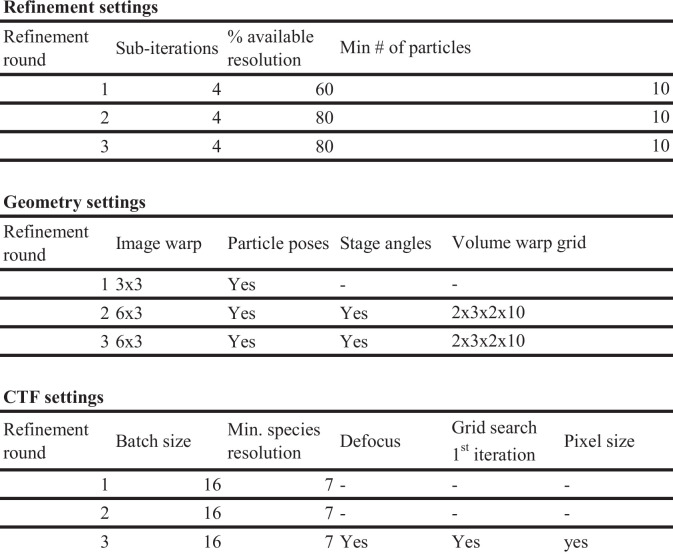
M refinement procedure and parameters

**Extended Data Table 4:**
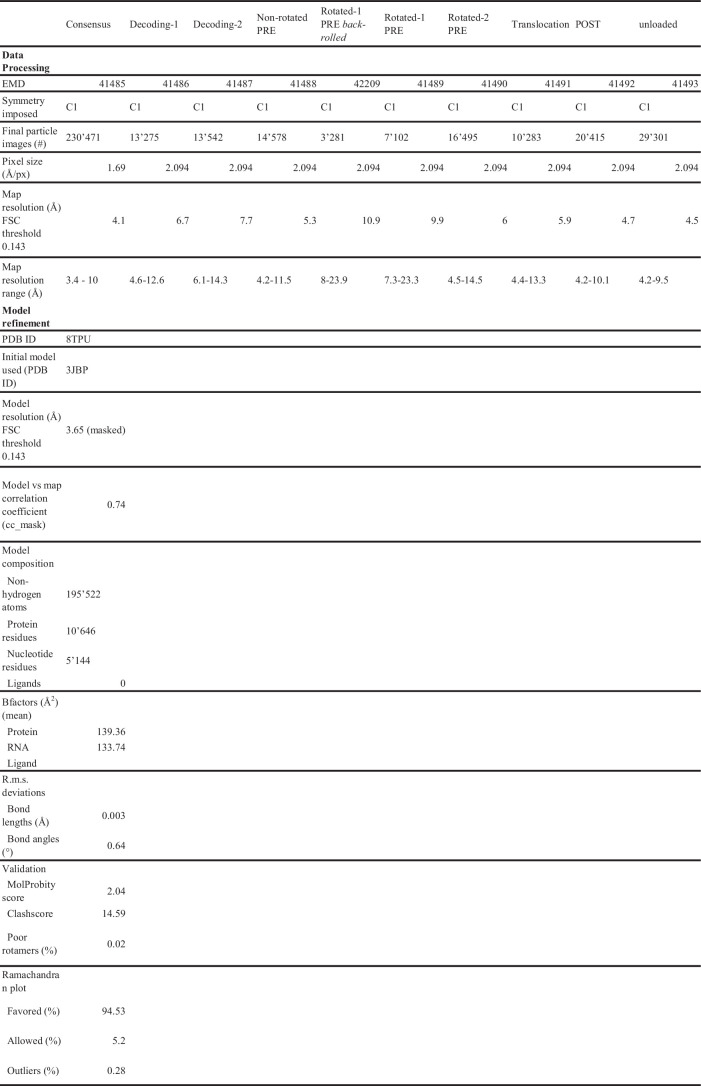
Final reconstructions of translation intermediates of STA maps and Model building and refinement of STA

## Data Availability

EM maps were deposited to the EM Data Bank under accession numbers EMD-41485, EMD-41486, EMD-41487, EMD-41488, EMD-41489, EMD-41490, EMD-41491, EMD-41492, EMD-41493, EMD-41494, EMD-42209, EMD-42210 and EMD-42211. Models were deposited to the PDB under accession numbers 9BUP, 9BUQ, 9BUS, 9BUT, 9BUU and 8TPU. Raw and processed MS data were uploaded to the MassIVE repository with accession number MSV00009987. Raw MS data for DIA thermal PISA experiments are available at JPOSTrepo, a member of ProteomeXchange Consortium, under accession numbers JPST003546 and PXD059612. Source data are provided with this paper.
